# Muscle-dependent regulation of adipose tissue function in long-lived growth hormone-mutant mice

**DOI:** 10.18632/aging.103380

**Published:** 2020-05-28

**Authors:** Xinna Li, Jacquelyn A. Frazier, Edward Spahiu, Madaline McPherson, Richard A. Miller

**Affiliations:** 1 Department of Pathology, University of Michigan School of Medicine, Ann Arbor, Michigan 48109, USA; 2 College of Literature, Sciences, and The Arts, University of Michigan, Ann Arbor, Michigan 48109, USA; 3 University of Michigan Geriatrics Center, Ann Arbor, Michigan 48109, USA

**Keywords:** aging, growth hormone, uncoupling protein 1 (UCP1), adipose tissue, inflammation

## Abstract

Altered adipose tissue may contribute to the longevity of Snell dwarf and growth hormone receptor (GHR) knock-out mice. We report here that white (WAT) and brown (BAT) fat have elevated UCP1 in both kinds of mice, and that adipocytes in WAT depots turn beige/brown. These imply increased thermogenesis and are expected to lead to improved glucose control. Both kinds of long-lived mice show lower levels of inflammatory M1 macrophages and higher levels of anti-inflammatory M2 macrophages in BAT and WAT, with correspondingly lower levels of TNFα, IL-6, and MCP1. Experiments with mice with tissue-specific disruption of GHR showed that these adipocyte and macrophage changes were not due to hepatic IGF1 production nor to direct GH effects on adipocytes, but instead reflect GH effects on muscle. Muscles deprived of GH signals, either globally (GKO) or in muscle only (MKO), produce higher levels of circulating irisin and its precursor FNDC5. The data thus suggest that the changes in adipose tissue differentiation and inflammatory status seen in long-lived mutant mice reflect interruption of GH-dependent irisin inhibition, with consequential effects on metabolism and thermogenesis.

## INTRODUCTION

Adipose tissue constitutes the largest endocrine organ in mammals and plays a crucial role in regulating energy homeostasis [1]. Adipose tissue modulates energy regulation both by endocrine secretion and by modification of blood nutrient concentrations and quality. Reciprocally, adipocyte tissue activity itself depends on the hormonal and nutritional influences that cause fat cells to either store excess nutrients as intracellular lipid, or release stored energy as heat [2]. Age-associated changes in this process can have significant physiological effects.

With the onset of aging, adipose tissue undergoes dramatic changes in content, distribution, and function. Fat distribution shifts from subcutaneous fat to visceral fat storage, and more triglycerides are stored in internal organs, such as the liver, heart, kidney, and muscle. In the process, heat production of adipose tissue is reduced [3, 4]. In turn, the increased visceral fat, increased triglyceride storage, and reduced stored energy have systemic metabolic effects that promote type 2 diabetes, inflammatory diseases, and insulin resistance, with effects on obesity, cardiovascular diseases, cancer, and lifespan [5–7]. Long-lived mutant mice, such as Ames dwarf, Snell dwarf and GKO mice, have increased percent body fat and abnormal fat distribution, with preservation of subcutaneous and relatively less visceral fat compared to controls [8–11], raising the idea that altered function of adipose tissue within these mice may contribute to their insulin sensitivity, longevity and disease resistance. To delineate the effects of GH on specific tissues, we evaluated adipose tissue in mice with global disruption of GHR (GKO mice), as well as mice with disruption of GHR in liver (LKO), muscle (MKO, or fat (FKO). Derivation and physiological characteristics of these four mouse models have been described in these studies [12–15].

Based on the structure and function of adipocytes and their surrounding stroma, adipose tissue is divided into two categories, white adipose tissue (WAT) and brown adipose tissue (BAT). WAT is mainly composed of unilocular adipocytes. Its function is to store excess energy in the form of triglycerides for future use. BAT consists of small, multilocular adipocytes and is responsible for dissipating energy in the form of heat through non-shivering thermogenesis. Newborns have a much higher proportion of BAT than adults, and the percentage of BAT gradually drops with age [16]. BAT is mainly distributed between the scapula, on the back of the neck, and around the kidneys [5, 17, 18]. High mitochondrial density causes BAT to appear darker than WAT. The mitochondrial inner membrane of brown fat cells is rich in uncoupling protein 1 (UCP1), a thermogenic protein. UCP1 uncouples mitochondrial oxidative phosphorylation, and increases metabolism of free fatty acids; the energy thus generated is released in the form of heat [19, 20]. Relatively recent work has documented a UCP1-positive fat cell within WAT. Cold stimulation or β3-adrenergic receptor agonists can increase the number of UCP1-positive fat cells in WAT depots, producing a cell with a BAT-like phenotype, referred to as beige or “brite” (brown in white) fat [21]. Similar to BAT, these beige cells have a multilocular fat droplet structure, and a high mitochondrial count, and they express brown fat-specific genes, such as UCP1. Together, BAT and beige cells are able to carry out rapid thermogenic responses and influence an organism’s overall capacity to expend energy [22, 23].

Adipose tissue also plays an important role as an immuno-regulatory organ, influencing the activity of macrophages, T cells, B cells, mast cells, dendritic cells and neutrophils [24]. The inflammatory response of adipose tissue is mainly regulated by macrophages. M1 macrophages produce pro-inflammatory cytokines, such as TNF-α, interleukin IL-6 and MCP-1 [25]. In contrast, M2 macrophages are anti-inflammatory and help to maintain tissue homeostasis [25, 26]. Adipose tissue inflammation therefore reflects the balance between pro-inflammatory M1 and anti-inflammatory M2 macrophage subtypes [25]. Aging is associated with chronic low-grade adipose inflammation, linked to insulin resistance. Particularly in obese individuals, the inflammatory response caused by M1 macrophages contributes to age-related health issues and insulin resistance, while M2 macrophages are characteristic of slender, healthy individuals [27–29]. Thus, M1/M2 macrophage polarization provides an index of this age-related inflammation [30, 31]. In principle, delay or reversal of M1/M2 macrophage polarization might contribute to the insulin sensitivity, disease resistance, and longevity of Ames, Snell, or GKO mice, but no data on this point are yet available.

Aging impairs thermogenic capacity of BAT [32–34], and an anti-aging intervention (calorie restriction) mitigates age-associated decline in brown/beige fat [35]. Long-lived Ames dwarf and GKO mice have enlarged BAT depots, as well as increased UCP1 mRNA expression [36–39], but evaluation of thermogenic capacity and differentiation of WAT has not yet been conducted, nor are there prior data on whether changes in fat cell differentiation reflect direct effects of GH, effects mediated by IGF-1, or other indirect endocrine-driven pathways.

Here we have used GKO mice, Snell dwarf mice, and mice with disruption of GHR in liver, muscle, or fat (respectively LKO, MKO, and FKO) to shed light on the endocrine control of thermogenesis in WAT and BAT, their links to macrophage polarization and inflammation, and the role of muscle-dependent signals in regulation of fat cell differentiation.

## RESULTS

### The global deletion of GHR promotes the induction of UCP1 expression in both WAT and BAT

**Figure 2:**
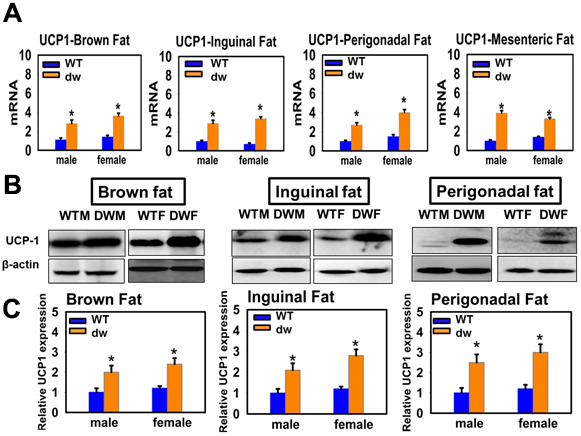
**Expression of UCP1 in adipose tissue of Snell Dwarf mice (dw).** (**A**) RNA was isolated from brown fat, mesenteric, inguinal and perigonadal adipose tissues of 24-week-old littermate control (WT) mice and Snell Dwarf mice (dw). mRNA levels of UCP1 were measured by qRT-PCR. Data (mean ± SEM; *n* = 4) were normalized by the amount of GAPDH mRNA and expressed relative to the corresponding male WT value. *P < 0.05 versus WT. (**B**) Cell lysate was prepared from brown fat, inguinal and perigonadal adipose tissues of 24-week-old WT and dw mice, and protein levels of UCP1 were measured by western blotting. Representative gel images are shown. (**C**) Relative protein expression was normalized to β-actin levels. Values are mean ±SEM (n = 4).

UCP1 uncouples energy expenditure in AT, is higher in BAT [23], and serves as a marker for WAT browning. UCP1 mRNA levels were increased almost 4-fold in BAT of GKO males and females (*P*<0.05) (Figure 1A). UCP1 protein levels were also significantly higher in BAT of GKO males and females (*P*<0.05) (Figure 1B, 1C). UCP1 mRNA levels were also 2.5x higher in each of the three tested WAT depots (inguinal, perigonadal and mesenteric), in GKO males and females (*P*<0.05) (Figure 1A), as were UCP1 protein levels (Figure 1B, 1C). UCP1 mRNA and protein levels were also higher in Snell dwarf mice (both male and female) relative to WT controls (Figure 2), suggesting that lower GH-mediated signaling augments UCP1 gene expression in BAT and WAT of both varieties of these long-lived mutant mice.

**Figure 1:**

**Effects of global deletion of Growth Hormone Receptor (GKO mouse) on the expression of UCP1 in adipose tissue.** (**A**) Total RNAs were isolated from interscapular (brown fat), mesenteric, inguinal and perigonadal adipose tissues of 24-week-old wild type littermate control mice (WT) and GKO mice. mRNA levels of UCP1 (brown and beige fat marker) were measured by qRT-PCR. Data (mean ± SEM; *n* = 4) were normalized by the amount of GAPDH mRNA and expressed relative to the corresponding male WT value. *P < 0.05 versus WT. (**B**) Cell lysate was prepared from interscapular (brown fat), inguinal and perigonadal adipose tissues of 24-week-old WT and GKO mice. Protein levels of UCP1 (brown and beige fat marker) were then measured by western blotting. Representative gel images are shown. (**C**) Relative protein expression was normalized to β-actin levels. Values are mean ±SEM (n = 4).

### Global deletion of GHR (GKO) results in a reduction in adipocyte size and an increase in adipocyte number in BAT and WAT

Adipocyte cell size determines the insulin reactivity of the adipose tissue; the smaller the fat cells, the more sensitive the tissue is to insulin [40, 41]. Since GKO mice are known to be insulin-sensitive, we evaluated adipocyte cell size and number in BAT and WAT of GKO and control adults. BAT of GKO mice contained an excess of smaller adipocytes (*P*<0.05) (Supplementary Figure 1A, 1C), and the same is true of inguinal WAT (*P*<0.05) (Supplementary Figure 1B, 1C). Consistent with the size difference, adipocyte cell numbers were elevated in both BAT and WAT of GKO mice (*P*<0.05) (Supplementary Figure 1D.).

### Liver-specific deletion of GHR (LKO) has no effect on the size of adipocytes or the number of adipocytes in BAT and WAT.

LKO mice have 90% lower levels of serum IGF-1, and thus provide a test of whether effects of global deletion of GHR are mediated by IGF-1 or other liver-specific GH-dependent pathways [42, 43]. We found that LKO mice, which are not long-lived [44], did not differ from littermate controls in adipocyte size or number in BAT or inguinal WAT (Supplementary Figure 2). These results suggested that the changes in fat cell size and number in GKO mice do not result from or depend on changes in serum IGF-1.

### Effects of tissue-specific deletion of GHR on UCP1 expression in WAT and BAT

**Figure 3:**
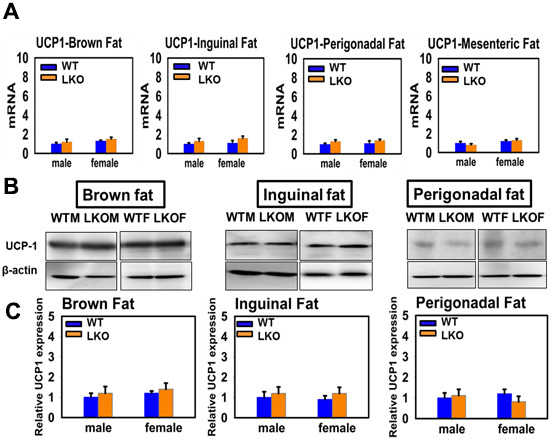
**Effects of liver-specific deletion of GHR (LKO mice) on the expression of UCP1 in adipose tissue.** (**A**) Total RNAs were isolated from brown fat, mesenteric, inguinal and perigonadal adipose tissues of 24-week-old WT mice and LKO mice. mRNA levels of UCP1 were measured by qRT-PCR. Data (mean ± SEM; *n* = 4) were normalized by the amount of GAPDH mRNA and expressed relative to the corresponding male WT value. *P < 0.05 versus WT. (**B**) Cell lysate was isolated from interscapular (brown fat), inguinal and perigonadal adipose tissues of 24-week-old WT mice and LKO mice, and protein levels of UCP1 were measured by western blotting. Representative gel images are shown. (**C**) Relative protein expression was normalized to β-actin levels. Values are mean ±SEM (n = 4).

We measured UCP1 mRNA and protein levels in adipose tissues of mice with tissue-specific GHR deletion (LKO, MKO and FKO). LKO males and females showed no effects on expression of UCP1 mRNA or protein in BAT or in any of the three tested WAT depots (Figure 3). These results suggest that the low circulating IGF-1 seen in GKO mice is not sufficient for the observed alterations in BAT and WAT UCP1.

**Figure 4:**
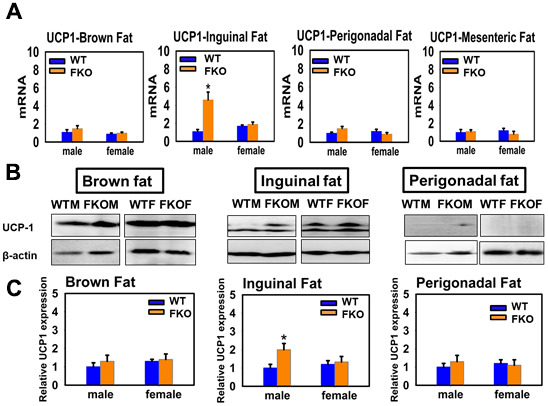
**Effects of fat-specific deletion of GHR (FKO mice) on the expression of UCP1 in adipose tissue.** (**A**) Total RNAs were isolated from brown fat, mesenteric, inguinal and perigonadal adipose tissues of 24-week-old WT mice and FKO mice. mRNA levels of UCP1 were measured by qRT-PCR. Values were normalized by the amount of GAPDH mRNA and expressed relative to the corresponding male WT value. *P < 0.05 versus WT. (**B**) Cell lysate was isolated from interscapular (BAT), inguinal and perigonadal adipose tissues of 24-week-old WT mice and FKO mice, and protein levels of UCP1 were measured by western blotting. Representative gel images are shown. (**C**) Relative protein expression was normalized to β-actin levels. Values are mean ±SEM (n = 4).

Similarly, disruption of GHR in fat tissue fails to replicate the effects of global KO of the GHR (Figure 4). UCP1 mRNA is not altered in BAT or in mesenteric or perigonadal fat in either sex, and UCP1 protein, similarly, is unaffected by FKO in BAT or perigonadal fat. Inguinal fat shows a sex-specific effect: FKO has no effect in females, but FKO males resemble GKO males in their higher levels of UCP1 protein and mRNA.

**Figure 5:**
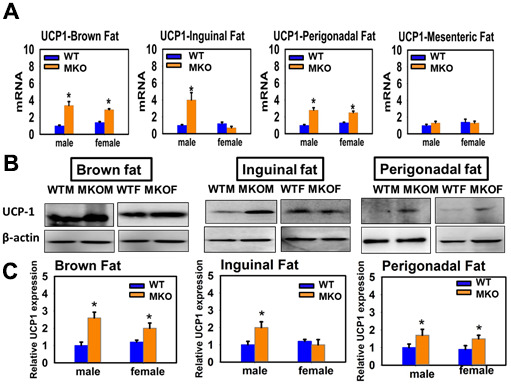
**Effects of muscle-specific deletion of GHR (MKO mice) on the expression of UCP1 in adipose tissue.** (**A**) Total RNA was isolated from brown fat, mesenteric, inguinal and perigonadal adipose tissues of 24-week-old WT mice and MKO mice. mRNA levels of UCP1 were measured by qRT-PCR. Values were normalized by the amount of GAPDH mRNA and expressed relative to the corresponding male WT value. *P < 0.05 versus WT. (**B**) Cell lysate was isolated from interscapular (brown fat), inguinal and perigonadal adipose tissues of 24-week-old WT mice and MKO mice, and protein levels of UCP1 were measured by western blotting. Representative gel images are shown. (**C**) Relative protein expression was normalized to β-actin levels. Values are mean ±SEM (n = 4).

In contrast, muscle-specific KO of GHR mimics most of the effects of global KO on fat cell UCP1 (Figure 5). UCP1 is elevated, for protein and mRNA, in BAT and in perigonadal WAT of MKO mice of both sexes, as well as in male (but not female) inguinal WAT. For mesenteric WAT, only mRNA data were available, and this tissue did not show any alteration of UCP1 mRNA. This surprising set of observations, together with the lack of effect of FKO, suggests that GH modulation of UCP1 levels in fat represents a GH-dependent effect of skeletal muscle on fat cell differentiation, with male-specific changes in inguinal WAT an exception to this pattern.

### Global deletion of GHR modifies macrophage M1-M2 polarization and reduces adipose inflammation

**Figure 6:**
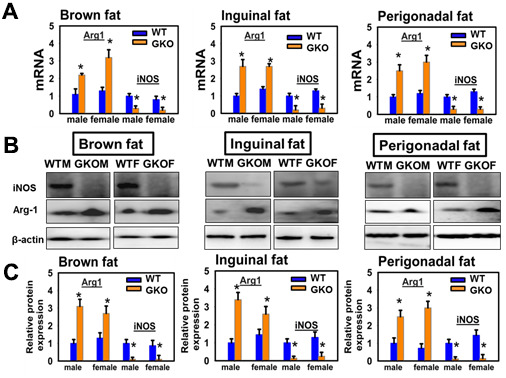
**Effects of global deletion of GHR (GKO mice) on adipose tissue macrophage infiltration and macrophage M1-M2 polarization.** (**A**) Quantitative RT-PCR analysis of total RNA isolated from interscapular (brown fat), inguinal and perigonadal adipose tissues of 24-week-old WT and GKO mice for M1 macrophage markers (iNOS) and M2 macrophage markers (Arg1) mRNAs. Data (mean ± SEM; *n* = 4) were normalized by the amount of GAPDH mRNA and expressed relative to the corresponding male WT value. *P < 0.05, **P < 0.01 versus WT. (**B**) Cell lysate was isolated from interscapular (brown fat), inguinal and perigonadal adipose tissues of 24-week-old WT and GKO mice. The protein levels of iNOS and Arg1 were measured by western blotting. (**C**) Relative protein expression was normalized to β-actin levels. Values are mean ±SEM (n = 4).

Immunoblotting data revealed lower levels of the M1 marker iNOS and elevation of the M2 marker Arg1 in BAT and in inguinal and perigonadal WAT of both male and female GKO mice (Figure 6). The observations for Arg1 and iNOS protein reflected parallel changes in the corresponding mRNAs. Snell dwarf mice showed the same shift from M1 to M2 polarization (Supplementary Figure 3). We also used immunohistochemistry (IHC) to confirm the observations for GKO mice, and noted a decrease in CD80^+^ macrophages and crown-like structures (CLSS) in brown and inguinal adipose tissue of GKO mice (*P*<0.05) (Supplementary Figures 4, 5). IHC staining further showed that GKO mice had significantly increased numbers of CD163^+^, F4/80^+^ M2 macrophages (*P*<0.05) (Supplementary Figures 4, 5). The changes in CD163 and CD80 were also reflected at the level of mRNA.

**Figure 7:**
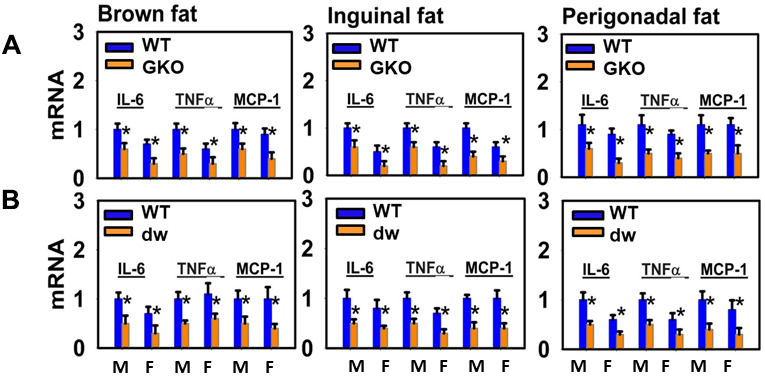
**Adipose tissue macrophage infiltration and macrophage M1-M2 polarization of long-lived mice (DW and GKO).** (**A**) Quantitative RT-PCR analysis of total RNA isolated from brown fat, inguinal and perigonadal adipose tissues of 24-week-old GKO mice and WT littermate mice for IL-6, TNFα, MCP-1 mRNAs. Values were normalized by the amount of GAPDH mRNA and expressed relative to the corresponding male WT value. *P < 0.05 versus WT. (**B**) Quantitative RT-PCR analysis of total RNA isolated from brown fat, inguinal and perigonadal adipose tissues of 24-week-old dw mice and WT mice for IL-6, TNFα, MCP-1 mRNAs. Data (mean ± SEM; *n* = 4) are expressed relative to the corresponding male WT value. *P < 0.05 versus WT.

Activated M1 cells secrete pro-inflammatory cytokines such as TNF-α, IL-6 and monocyte chemotactic protein-1 (MCP-1), thereby blocking the action of insulin in fat cells [45, 46]. We found that expression of mRNA for all three cytokines was significantly decreased in BAT and WAT (inguinal fat and perigonadal fat) of GKO males and females (*P*<0.05) (Figure 7). Thus, global disruption of GHR leads to increases in the ratio of M2/M1 cells as well as lower levels of cytokine production in BAT and WAT. mRNA for each of these cytokines is also significantly elevated in BAT and WAT of Snell dwarf mice (Figure 7). Thus, the cytokine mRNA data are entirely consistent with the results from immunoblotting for iNOS and Arg1 (Figure 6) and the IHC results. All of these changes indicate a shift from inflammatory M1 to anti-inflammatory M2 macrophages in GKO mice.

### Effects of organ-specific deletion of GHR on macrophage M1-M2 polarization and cytokine production in WAT and BAT

**Figure 8:**
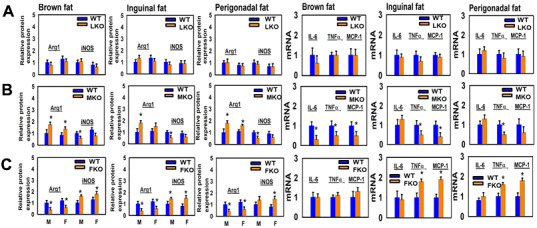
**Adipose tissue macrophage infiltration and macrophage M1-M2 polarization of tissue-specific GHR KO mice (LKO, MKO and FKO).** The three left panels show relative protein expression (Arginase1 and iNOS) in brown fat, inguinal and perigonadal adipose tissues of 24-week-old LKO (**A**), MKO (**B**), and FKO (**C**) was normalized to β-actin levels. Values are mean ±SEM (n = 4). *P < 0.05 versus WT. M = males; F = females. The three right panels show quantitative RT-PCR analysis of total RNA isolated from brown fat, inguinal and perigonadal adipose tissues of 24-week-old LKO (**A**), MKO (**B**), and FKO (**C**) mice and WT mice for IL-6, TNFα, MCP-1 mRNAs. Data (mean ± SEM; *n* = 4) were normalized by the amount of GAPDH mRNA and expressed relative to the corresponding male WT value. *P < 0.05 versus WT.

To follow our observation that alterations in adipose tissue UCP1 were regulated by GHR expression in muscle, rather than fat or liver tissue, we next evaluated markers of macrophage polarization (Arg1 and iNOS) in BAT, and inguinal and perigonadal WAT, of mice with disruption of GHR in liver, muscle or fat (Figure 8, first three columns). The pattern seen in GKO mice – increased Arg1 and decreased iNOS – was seen only in the MKO mice (Supplementary Figure 6). LKO mice did not have significant change in Arg1 or iNOS in any of these three tissues (Supplementary Figure 6). Interestingly, the FKO mice showed significant changes in the opposite direction, with higher levels of iNOS and lower levels of Arg1 protein (Supplementary Figure 6), suggesting a possible increase in the balance of inflammatory to anti-inflammatory macrophages. We also evaluated mRNA levels for three cytokines, IL-6, TNFα, and MCP1 in the same tissues (Figure 8, last three columns). Consistent with the data on M1 and M2 marker proteins (and Supplementary Figure 7), there were no significant changes in cytokine mRNAs in the LKO mice, but MKO showed significant declines in IL-6 (BAT only), in TNFα, and in MCP1. The FKO mice had significant increases in TNFα and MCP1 in the two WAT depots, suggesting increased inflammatory activity, consistent with the data on Arg1 and iNOS in these mice, and opposite in direction to the results seen in GKO and MKO mice.

### Tissue-specific GH control of FNDC5/irisin, a mediator of adipose tissue differentiation

**Figure 9:**
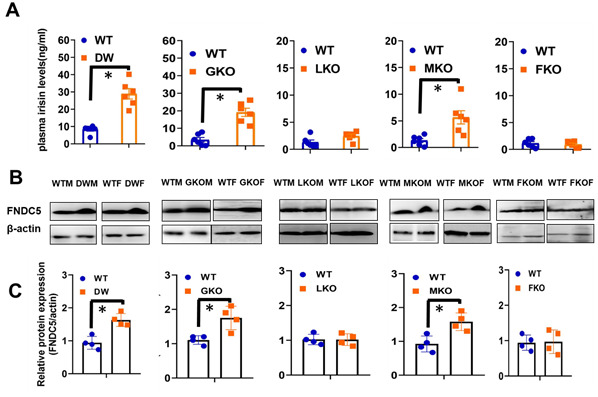
**Plasma irisin levels and expression of FNDC5 in muscle tissue of WT and mutant mice (DW, GKO, LKO, MKO and FKO).** (**A**) Irisin content was measured by ELISA assay on plasma samples of 24-week-old WT and mutant mice model (DW, GKO, LKO, MKO and FKO). Data are shown as mean ± SEM for each group (n = 6). *P < 0.05 versus WT. (**B**) Cell lysate was prepared from gastrocnemius muscle of 24-week-old WT and mutant mice (DW, GKO, LKO, MKO and FKO), and protein levels of FNDC5 were measured by western blotting. Representative gel images are shown. (**C**) Relative protein expression was normalized to β-actin levels. Values are mean ±SEM (*n* = 4). *P < 0.05 versus WT.

Despite some controversies [47–50], there is evidence that circulating irisin, a cleavage product of the transmembrane protein FNDC5, communicates exercise-triggered, PGC-1α-regulated changes in muscle cell status to various fat depots, stimulating UCP1, thermogenesis, and differentiation of white to brown or beige adipocytes [51–53]. Irisin is associated with reduction of pro-inflammatory cytokines (TNFα, IL-1β, IL-6, MCP-1) and promotes secretion of anti-inflammatory cytokines (IL-10, IL-4, IL-13) in adipose tissue [54–56]. We therefore hypothesized that plasma irisin levels and muscle FNDC5 might underlie the effects of Snell and GHR mutations, in GKO and MKO mice, on UCP1 and markers of white-to-beige transition noted above. We found increased levels of plasma irisin in Snell dwarf, GKO, and MKO mice, but not in LKO or FKO strains (Figure 9, top panels). Consistent with this hypothesis, muscle tissue from Snell dwarf, GKO, and MKO mice had higher levels of FNDC5, the precursor of irisin, but there were no changes in FNDC5 protein in muscle of LKO or FKO mice.

## DISCUSSION

Low insulin and glucose levels, and reduced body temperature, are characteristic of long-lived Ames dwarf, Snell dwarf, and GKO mice [57, 58], and it has been proposed that these traits could contribute to the extended healthy life span in these mice. Conti and his colleagues reported that reduction of the core body temperature in Hcrt-UCP2 transgenic mice, which are engineered to overexpress UCP2 in hypocretin neurons (Hcrt-UCP2), leads to a significant increase of life span [59]. There is also evidence that lower body temperature is associated with increased longevity in humans [60]. Calorie restriction (CR), which extends longevity, also leads to decrease in body temperature in mice [61].

Ames dwarf, Snell dwarf and GKO mice exhibit an increase in percentage of body fat, but the distribution of fat mass is different from that seen in littermate controls, with disproportionate increases in the subcutaneous depots and lower levels of mesenteric fat [9, 37, 62]. Aging in control mice often leads to obesity and insulin resistance, but GKO and Snell dwarf mice maintain a youthful metabolic state: lean, insulin-sensitive, with high resting metabolic rate [63, 64]. In rats, surgical removal of visceral fat leads to increases in lifespan not seen in animals from which similar amounts of subcutaneous fat has been removed [65], suggesting heterogeneity in the actions of different fat depots.

UCP1 mRNA has been shown to be elevated in BAT of GKO and Ames dwarf mice [36, 37], and the amount of BAT is elevated in GKO mice [37]. Our own data on UCP1 confirm the report on GKO mice, replicate the Ames data with our findings on Snell dwarf mice, and show that the elevation in mRNA leads to corresponding changes in UCP1 protein. More importantly, we show further that UCP1 is elevated in three varieties of WAT, including both subcutaneous and intra-peritoneal depots. Thus, disruption of GH signals not only increases thermogenic capacity of BAT, but it also converts WAT cells to beige/brite adipocytes, with elevated UCP1 and restructuring of cell size and shape revealed by our IHC data.

Our results add three further insights to the developing model of how GH signals regulate fat depots and metabolism in directions likely to contribute to delayed aging and extended lifespan in Snell and GKO mice.

First, we find that the conversion of WAT to beige adipose tissue is accompanied by elevation of the numbers of anti-inflammatory M2 macrophages and parallel decline in the numbers, and cytokine production, by pro-inflammatory M1 macrophages. Such an increase in the ratio of M2/M1 cells is associated with retention of youthful metabolic status [66–68], and, conversely, lower M2/M1 ratios are characteristic of many varieties of metabolic disease. Our results are consistent with a previous report that IL6 is diminished in plasma and epididymal fat of Ames dwarf and GKO mice [64, 69], together with increases in adiponectin in the Ames mice.

We do not know if the change in M2/M1 ratio in these low-GH/GHR mice leads to UCP1 upregulation and conversion of WAT to beige tissue, or if the conversion to beige tissue promotes M2 accumulation and M1 loss. It is also possible that each of these changes could be an independent consequence of diminished GH tone in some other, unknown tissue. The up-regulation of brown fat and beige fat thermogenesis is typically inversely correlated with the expression of inflammatory genes [70]. Recently, several studies have reported that anti-inflammatory M2 macrophages within AT play crucial roles in the regulation of BAT thermogenic activity and WAT conversion to beige status [71–73]. There is also evidence that anti-inflammatory macrophages (M2) are directly involved in promoting BAT thermogenesis [74]. M2 macrophages in WAT from cold-stimulated mice were also found to be involved in the WAT browning process [71, 74] Conversely, several signals have been found to originate in BAT and WAT that induce M2 macrophage polarization and recruitment, which then establish local positive feedforward mechanisms of fat beiging activation [72–76]. Thus, the direction of cause and effect linking macrophage polarization and beige conversion is not yet a settled matter. It would be of interest to evaluate mice in which GHR was disrupted in macrophages or their precursors, and to evaluate M2/M1 ratios in non-adipose tissues of long-lived mutant mice. Data on beige cells and on macrophage polarization in mice treated with drugs that extend lifespan would also be of interest in this connection.

Second, our results show that the alteration in adipose tissue UCP1 levels, beige cell differentiation, and macrophage polarization do not reflect direct effects of GH on fat cells themselves; nor do they reflect GH action mediated by IGF-1 produced by the liver. Instead, most of the changes in BAT and WAT seen in GKO mice can be mimicked by disruption of GHR in skeletal muscle cells (in MKO mice). Indeed, most of the changes in cytokine production and M2/M1 polarization seen in FKO mice are opposite in direction to those seen in GKO (and MKO) animals; FKO mice have lower ratios of M2/M1 and increased production of cytokines characteristic of M1 cells. FKO mice are larger than control mice, with increased mass of both WAT and BAT, and exhibit a decreased lifespan [14]; it is possible that increased inflammation in adipose or other tissues of FKO animals contribute to their early demise. Our results on cytokine mRNA in the adipose tissue are consistent with a prior report of diminished circulating IL6 in MKO mice [77]. We cannot rule out that alterations of other cellular signals in GKO mice, potentially mediated by secreted brain hormones or innervation of fat depots, may also be altered in GKO and Snell mice, but our data on MKO mice show that alteration of GH/GHR signals in muscle is sufficient to re-create many of the key changes seen in adipose tissue of GKO mice.

Lastly, our data suggest a mechanism by which GH/GHR disruption in muscle may lead to systemic changes in adipose tissue, i.e. production and secretion of irisin through cleavage of FNDC5 in muscle. Plasma irisin is elevated in Snell, GKO and MKO mice, but not in LKO or FKO animals. Similarly, FNDC5, from which irisin is generated as a cleavage product, is elevated in skeletal muscle of the same three varieties of mice. The simplest model is one in which GH acts on muscle to depress FNDC5 levels, so that removal of this signal in Snell, GKO, or MKO mice increases FNDC5 with parallel increase in irisin levels in the plasma. Other models are possible, in which GH signals modulate FNDC5 or irisin production in non-muscle tissues, or control the rate of FNDC5 cleavage or irisin stability. Irisin has been shown to stimulate white adipose tissue beiging by increasing the expression of uncoupling protein-1 (UCP-1) [51]. We do not have evidence yet that it is irisin per se that leads to the changes in adipose tissue seen in GKO, MKO, and Snell mice, a point we will pursue in further studies.

To help further investigate the role that GH exerts on adipose tissue in vivo, three mouse lines with altered GH signaling in specific organs (liver, fat, and muscle) were also used to study GH’s role in adipose function. FKO mice have increased percentage of fat in all adipose depots. Adipose-specific ablation of the GHR gene (FKO) results in an obese phenotype, while liver-specific ablation of the GHR gene (LKO) does not [78]. MKO is reported to exhibit increased body adiposity [79]. It has also been previously reported that MKO mice have increased insulin sensitivity, as well as reduced adipose tissue macrophage infiltration. MKO mice have significant reductions in circulating IL-6 levels, an adipocytokine associated with obesity-induced systemic inflammation insulin resistance [77]. LKO does not affect lifespan, and MKO males were significantly longer lived than control males at Michigan but not at Ohio University [14], with no longevity effect of MKO on female lifespan seen at either test site. LKO mice, which have high GH and low circulating IGF-I levels, had a higher body fat percentage at early ages followed by lower body fat percentage in adulthood [12, 78].

Tested in two vivaria, FKO mice are somewhat shorter lived than littermate controls [14]. The FKO mice used in our study are based on the Fabp4 (aP2 promoter). Although this promoter was originally thought to target disruption to adipocytes, more recent work has shown aP2 expression in other tissues, including macrophages, hypothalamus, other CNS neurons, and peripheral tissues including muscle [80]. The Kopchick laboratory has more recently evaluated a different stock of mice (“AdGHRKO”) in which GH disruption is driven by the adiponectin promoter/enhancer [81], and found that these mice show aspects of metabolic health that are absent in the FKO mice we use. There is no published information about lifespan of the AdGHRKO stock. It will be of high interest to evaluate UCP1, macrophage polarization, and FNDC5/irisin biology in the AdGHRKO mice. It is possible that the changes we note in FKO mice, including increases in the M1/M2 ratio and increases in WAT TNFα and MCP1 (Figure 8), could contribute to the small decline in lifespan noted in FKO mice.

It is not yet clear how these changes – changes in adipocyte size, increased UCP1 levels, reduction in inflammatory status of macrophages in adipose tissues, lower cytokine production, and irisin production – contribute to the disease resistance and increases in healthy lifespan of GKO and Snell dwarf mice. Although insulin sensitivity is characteristic of these long-lived mutant mice, diabetes is seldom a cause of death in these stocks. Many other aspects of aging are delayed or decelerated in these mice, and most of the mice die of some form of neoplasia. Links between GH/GHR regulation of tissue function and the pace of aging are not yet clearly delineated, but our work suggests that systemic alteration of adipose tissue cellularity, composition, cytokine production and thermogenic function may be secondary to GH-dependent signals from muscle, and could represent one of the key pathways leading to long-lasting health in these mouse stocks.

## MATERIALS AND METHODS

### Mice

Snell dwarf (homozygous dw/dw) animals (and heterozygote controls) were bred as the progeny of (DW/J × C3H/HeJ)-dw/+ females and (DW/J × C3H/HeJ) F1-dw/dw males. Littermates with the (+/dw) genotype were used as controls. GH receptor knockout (GHRKO, here termed GKO) mice and littermate controls were bred from breeding stock originally generated by Dr. John Kopchick’s group at Ohio University as previously described [82]. The three tissue-specific GHR^−/−^ mouse lines were then produced by breeding GHR^flox/flox^ mice to one of three Cre-recombinase transgenic mouse lines, each acquired from the Jackson Laboratory (Bar Harbor, ME). The adipose tissue-specific GHR^−/−^ mouse line (“FKO”) was generated by breeding GHR^flox/flox^ mice to B6.Cg-Tg (Fabp4-cre) 1 Rev/J mice. Liver tissue-specific GHR^−/−^ mice (“LKO”) were generated by breeding GHR^flox/flox^ mice to B6.Cg-Tg (Alb-cre)21Mgn/J mice. Skeletal muscle-specific GHR^−/−^ mice (“MKO”) were generated by breeding the conditional GHR^flox/flox^ mice to B6.FVB (129S4)-Tg (Ckmm-cre) 5 Khn/J mice. All three Cre-recombinase transgenic mouse lines were previously backcrossed into the C57BL/6J strain; therefore, the resulting cre-lox tissue-specific mouse lines were a mix of C57BL/6J and C57BL/6N substrains. Breedings were coordinated in such a manner that all three tissue-specific mouse lines used were C57BL/6 with an ~62.5% “J” and 37.5% “N” substrain mixture. Breeding pairs for each line were shipped from Ohio University to the University of Michigan where they were maintained in the same C57BL/6 (62.5% J/37.5% N) substrain mixture for all studies described herein [83]. The experimental protocols were reviewed and approved by the University Committee on the Use and Care of Animals at the University of Michigan.

### RNA isolation and cDNA synthesis

BAT, inguinal WAT, perigonadal WAT and mesenteric WAT samples were taken from adult mice 4 – 6 months of age; about half of the mice used in each experiment were males, and we did not note any sex-specificity of the results obtained. Samples were homogenized utilizing the Bullet Blender from Next Advance (Averill Park, NY, USA). Adipose tissue total RNA was isolated from mouse livers using CarbonPrep Phenol/Trizol kit (Life Magnetics, Inc, Detroit, MI) according to the manufacturer’s instruction. The RNA was cleaned using the QiagenRNeasy mini RNA cleanup protocol (Qiagen, Valencia, CA). The concentration of total RNA was performed by measuring the absorbance of RNA sample solutions at 260 nm by using a Nanodrop ND-100. Total RNA (1.0 μg) was reverse transcribed using iScript cDNA reverse transcription kits (1708891; Bio-Rad, Hercules, CA) according to the manufacturer’s instructions.

### Quantitative real-time PCR

qPCR was performed using the Fast Start Universal SYBR Green Master Mix (Applied Biosystems, Foster City, CA). RT-PCR was performed using quantitative PCR systems (Applied Biosystems® 7500 Real-Time PCR Systems, Thermo Fisher Scientific, Waltham, MA, USA) with corresponding primers (Supplementary Table 1, Invitrogen). Glyceraldehyde-3-phosphate dehydrogenase (GAPDH) was simultaneously assayed as a loading control. The cycle time (CT) was normalized to GAPDH in the same sample. The expression levels of mRNA were reported as fold changes vs. littermate control. Data was analyzed using a ΔΔCT approach.

### Histological analysis and determination of adipocyte size using H&E staining

Immediately after removal, adipose tissues (BAT, inguinal WAT) were fixed overnight by immersion in 10% paraformaldehyde at room temperature. Tissues were dehydrated, embedded in paraffin, sectioned at 5 μm thickness, and stained with hematoxylin and eosin (H&E) to evaluate adipose morphology using a light microscope. 10 images were taken from different areas on each slide. To measure adipocyte size, ImageJ software was used by drawing an outline around each fat droplet-containing cell (i.e. each adipocyte) on each image. The area within and perimeter of the outline were determined using the ‘measure’ function in ImageJ (https://imagej.nih.gov/ij/)

### Immunohistochemical (IHC) analysis

Paraffin adipose tissue sections were cut at room temperature and then deparaffinized through the dewatering process. Subsequently, the sections were immunostained with an antibody against macrophage markers F4/80 (Abcam, Cambridge, MA, USA), CD80 (Abcam, Cambridge, MA, USA) for M1 macrophages, and CD163 (Abcam, Cambridge, MA, USA) for M2 macrophages at 4°C overnight. The sections were then washed for 10 min in 1% phosphate-buffered saline (PBS), and incubated at room temperature for 1 hour with biotinylated secondary antibody, PE-conjugated goat anti-rabbit IgG (Santa Cruz Biotechnologies) followed by the Vectastain Elite ABC kit (Vector Labs). A DAB Peroxidase Substrate Kit (Vector Labs) was used to visualize peroxidase reaction. The number of CD163^+^, CD80^+^ and F4/80^+^macrophages was quantified microscopically for each slide from 5-10 randomly chosen fields of five independent mice, as previously described [84]. All images were captured with a microscope (BX51, OLYMPUS, JAPAN) and analyzed by a blinded observer with ImageJ. Cell numbers were calculated from three randomly-selected microscopic fields, and three consecutive sections were analyzed for each mouse.

### Western blot analyses

Proteins from BAT, inguinal WAT, perigonadal WAT and mesenteric WAT were extracted after homogenization in Radio-Immunoprecipitation Assay Buffer (RIPA Buffer, Fisher Scientific, Pittsburgh, PA, USA) supplemented with Complete Protease Inhibitor Cocktail (Roche Inc.). Protein content was measured using a BCA assay (Fisher Scientific, Pittsburgh, PA, USA). The protein extracts were separated by SDS/PAGE on a 4–15% running gel, transferred to polyvinylidene difluoride membranes, and electro-transferred to an Immobilon-P Transfer Membrane (Millipore, Billerica, MA, USA) for immunoblot analyses. Membranes were blocked in Tris buffered saline containing 0.05% Tween20 (TBS-T) and 5% Bovine Serum Albumin (BSA) for 1 hour. After blocking, membranes were probed overnight with primary antibodies in TBS-T supplemented with 5% BSA with shaking at 4°C, followed by three 10 minute washes with TBS-T, incubation with secondary antibody for 1 hour, and three 10 minute washes with TBS-T. Membranes were then evaluated using an ECL Chemiluminescent Substrate (Fisher Scientific, Pittsburgh, PA, USA). The following antibodies were used: anti-UCP1 (Abcam, catalog no. 10983, 1:1000), anti-Arg1 (Abcam, 1:1000), anti-iNOS (Abcam, 1:1000), anti-β-actin (Santa Cruz Biotechnology, 1:1000), HRP-conjugated anti-mouse (GE Healthcare UK Limited, 1:2000) and anti-rabbit (GE Healthcare UK Limited, 1:5000). Quantification was performed using ImageJ software.

### Statistical analysis

The data are presented from multiple independent experiments. All data are presented as mean ± SEM. The Student’s two tailed *t*-test was used for comparisons of two experimental groups. *P* < 0.05 was regarded as significant.

### Data availability

The data that support the findings of this study are available from the corresponding author on request.

## Supplementary Material

Supplementary Figures

Supplementary Table 1
