# Supplementary material for: Muscle-dependent regulation of adipose tissue function in long-lived growth hormone-mutant mice
Source: Aging (Albany NY). 2020 May 28;12(10):8766–89. doi: 10.18632/aging.103380 (PMC7288969; doi:10.18632/aging.103380)
Supplement: Supplementary Figures [file aging-12-103380-s002..pdf]

SUPPLEMENTARY FIGURES

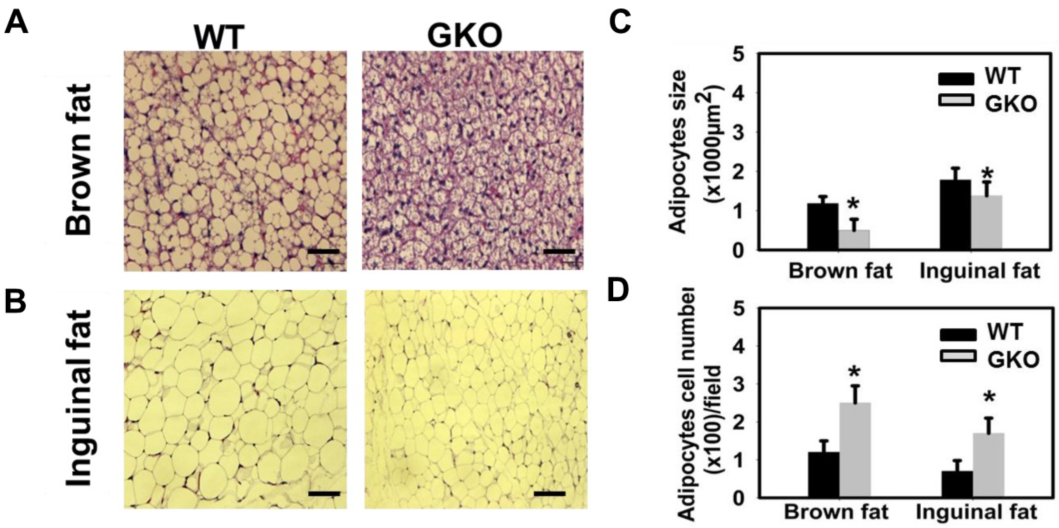

Supplementary Figure 1. GHRKO mice have reduced adipocyte cell size. 6-month old WT and GKO mice were used. (**A**) Representative images of adipose tissue stained with H&E. Hematoxylin and eosin (H & E) was performed on brown fat from wild-type (WT, n = 4) and GKO mice (n = 4) to assess morphology. Scale bars indicate 50 µM. (**B**) Representative images of adipose tissue stained with H&E. Hematoxylin and eosin (H & E) was performed on Inguinal WAT from wild-type (n = 4) and GKO mice (n = 4) to assess morphology. Scale bars indicate 50 µM. (**C**) Average size of the brown and white adipocytes of WT and GKO mice. $n = 4$, *, $p < 0.05$, WT vs. GKO mice. Adipocyte area was counted by ImageJ software. (**D**) Adipocyte cell number per field of brown fat and white adipocytes from WT and GKO mice. $n = 4$, *, $p < 0.05$, WT vs. GKO mice.

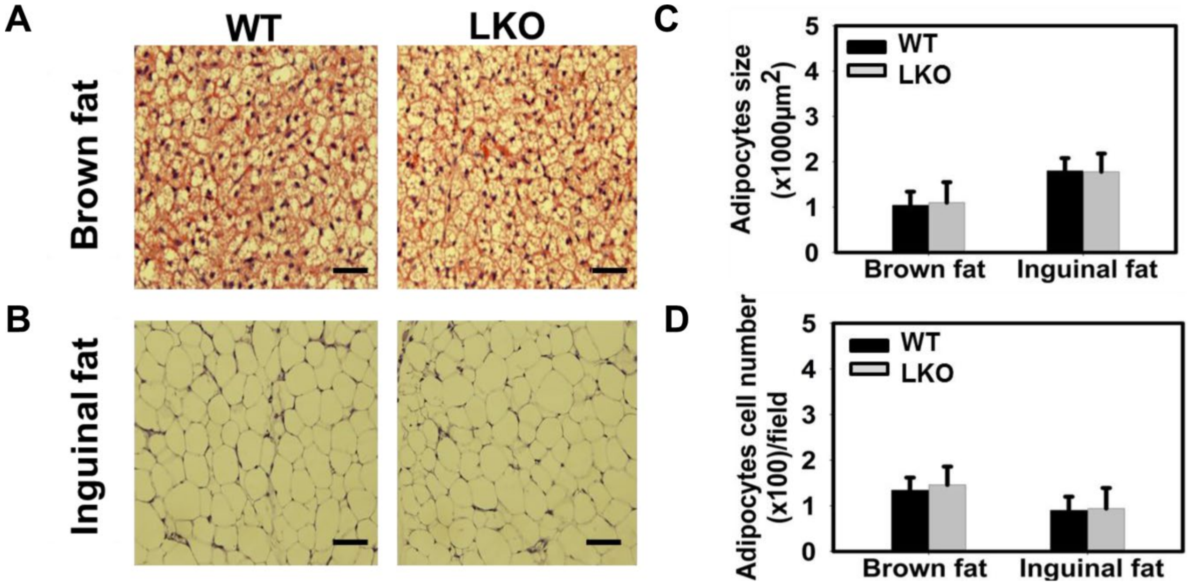

Supplementary Figure 2. Liver specific growth hormone receptor knock out mice (LKO) have similar adipocyte cell size with wide type mice. 6-month old WT and LKO mice were used. (**A**) Hematoxylin and eosin (H & E) was performed on brown fat from wild-type (WT, n = 4) and LKO mice (n = 4) to assess morphology. Scale bars indicate 50 µM. (**B**) Hematoxylin and eosin (H & E) was performed on Inguinal WAT from wild-type (WT, n = 4) and LKO mice (n = 4) to assess morphology. Scale bars indicate 50 µM. (**C**) Average size of the brown and white adipocytes of WT and LKO mice. $n = 4$, *, $p < 0.05$, WT vs. LKO mice. Adipocyte area was counted by ImageJ software. (**D**) Adipocyte cell number per field of brown fat and white adipocytes from WT and LKO mice. $n = 4$, *, $p < 0.05$, WT vs. LKO mice.

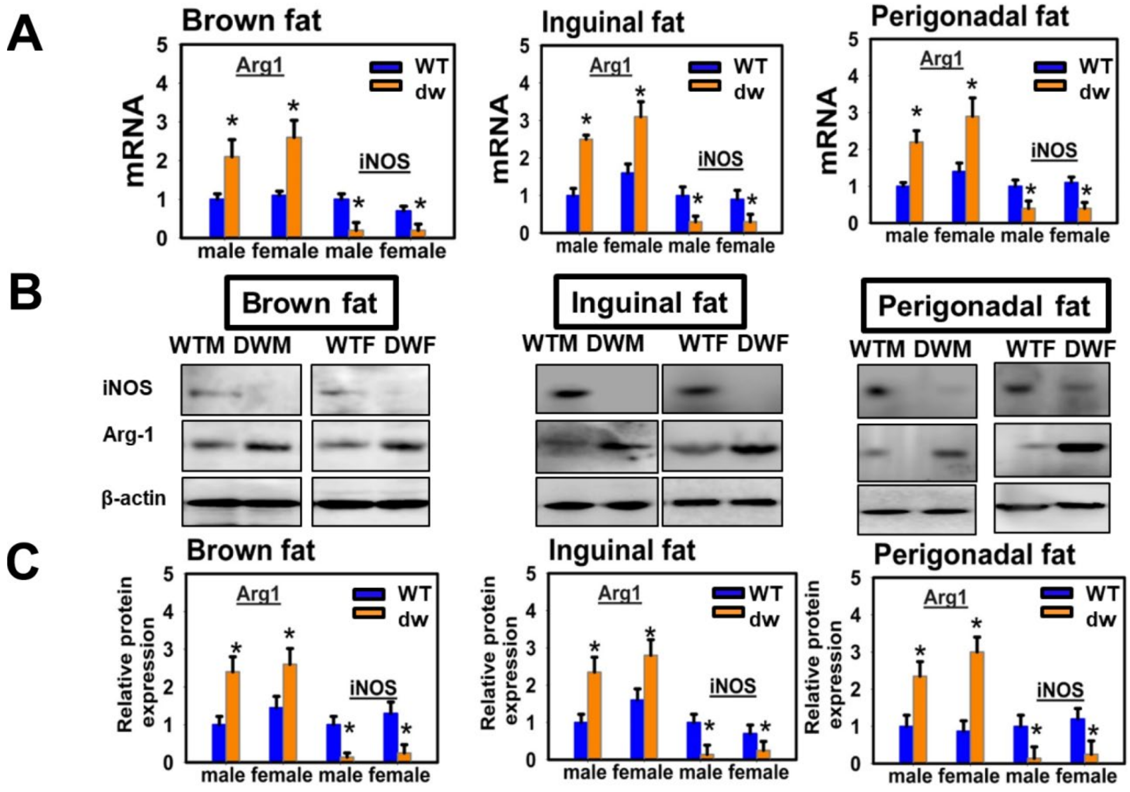

Supplementary Figure 3. Adipose tissue macrophage infiltration and macrophage M1-M2 polarization in Snell dwarf (dw/dw) mice. (**A**) Quantitative RT-PCR analysis of total RNA isolated from brown fat, inguinal and perigonadal adipose tissues of 24-week-old dw mice and WT mice for M1 macrophage markers (iNOS) and M2 macrophage markers (Arg1) mRNAs. Data (mean ± SEM; *n* = 4) were normalized by the amount of GAPDH mRNA and expressed relative to the corresponding male WT value. *$P < 0.05$, **$P < 0.01$ versus WT. (**B**) Cell lysate was isolated from interscapular (brown fat), inguinal and perigonadal adipose tissues of wt mice and dw mice. The protein levels of iNOS and Arg1 were measured by western blotting. (**C**) Relative protein expression was normalized to β-actin levels. Values are mean SEM (n = 4).

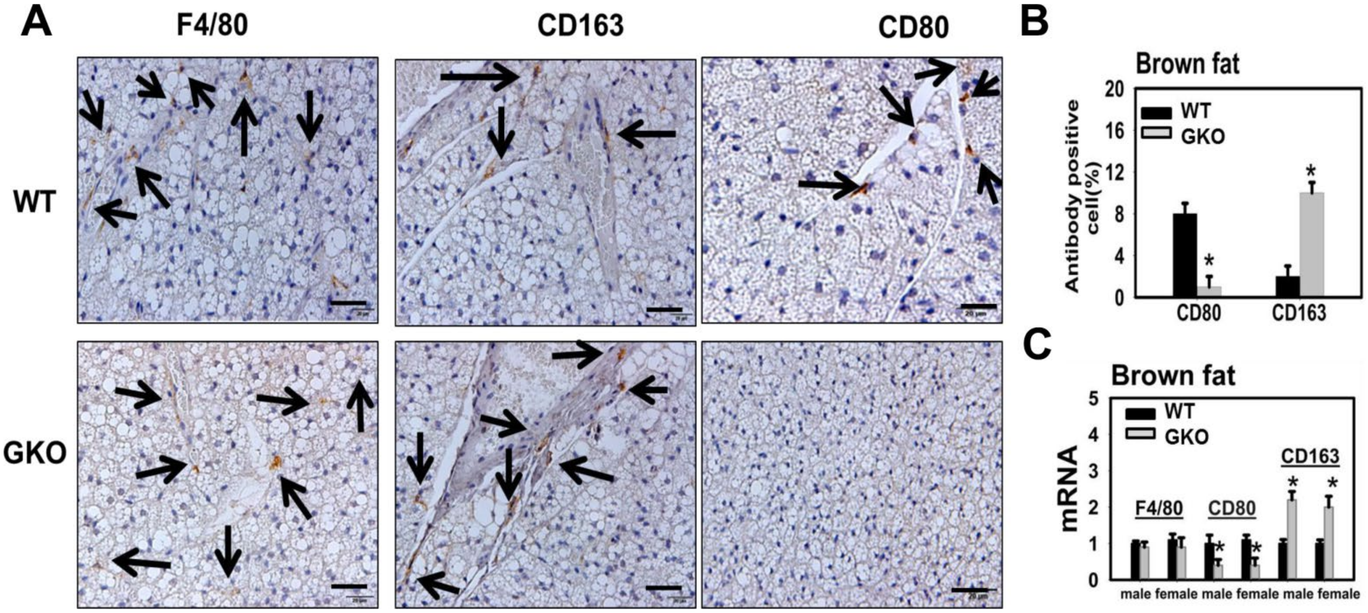

Supplementary Figure 4. Effects of Global deletion of Growth Hormone Receptor (GKO) on adipose tissue macrophage infiltration and macrophage M1-M2 polarization. (**A**) Representative images of brown fat sections from 24-week-old GKO mice show a lower expression of M1 macrophage markers (CD80) and a higher expression of M2 macrophage markers (CD163) compared to WT mice. Macrophages are stained brown with arrowheads. Scale bars: 50 µM. (**B**) Quantification of CD80-positive cells. $n = 4$, *, $p < 0.05$, WT vs. GKO mice. (**C**) Quantitative RT-PCR analysis of total RNA isolated from brown fat of 24-week-old GKO mice and WT mice for total macrophage marker (F4/80), M1 macrophage markers (CD80) and M2 macrophage markers (CD163) mRNAs. Data (mean ± SEM; $n = 4$) were normalized by the amount of GAPDH mRNA and expressed relative to the corresponding male WT value. *$P < 0.05$ versus WT.

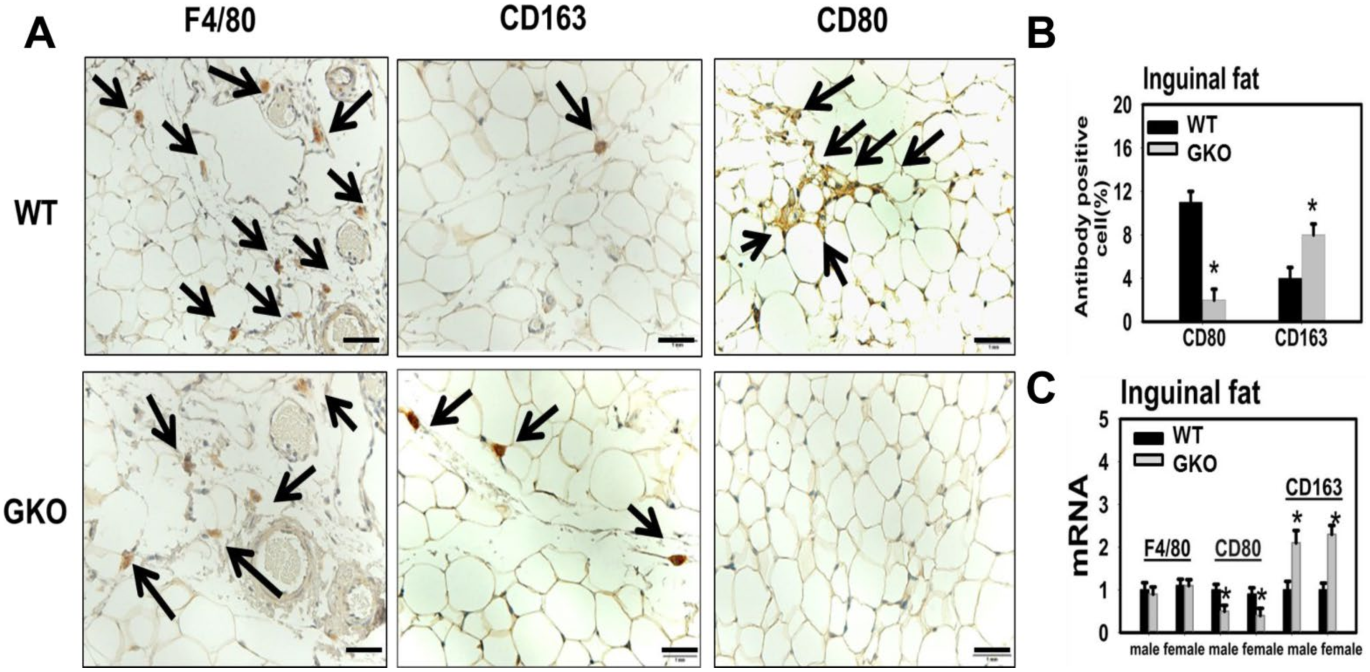

Supplementary Figure 5. Effects of Global deletion of Growth Hormone Receptor (GKO) on adipose tissue macrophage infiltration and macrophage M1-M2 polarization. (**A**) Representative images of white adipose tissue (inguinal fat) sections from 24-week-old GKO mice show a lower expression of M1 macrophage markers (CD80) and a higher expression of M2 macrophage markers (CD163) compared to WT mice. Macrophages are stained brown with arrowheads. Scale bars: 50 μM. (**B**) Quantification of CD80-positive cells. $n = 4$, *, $p < 0.05$, WT vs. GHRKO mice. (**C**) Quantitative RT-PCR analysis of total RNA isolated from inguinal adipose tissues of 24-week-old GKO mice and WT mice for total macrophage marker (F4/80), M1 macrophage markers (CD80) and M2 macrophage markers (CD163) mRNAs. Data (mean ± SEM; $n = 4$) were normalized by the amount of GAPDH mRNA and expressed relative to the corresponding male WT value. *$P < 0.05$ versus WT.

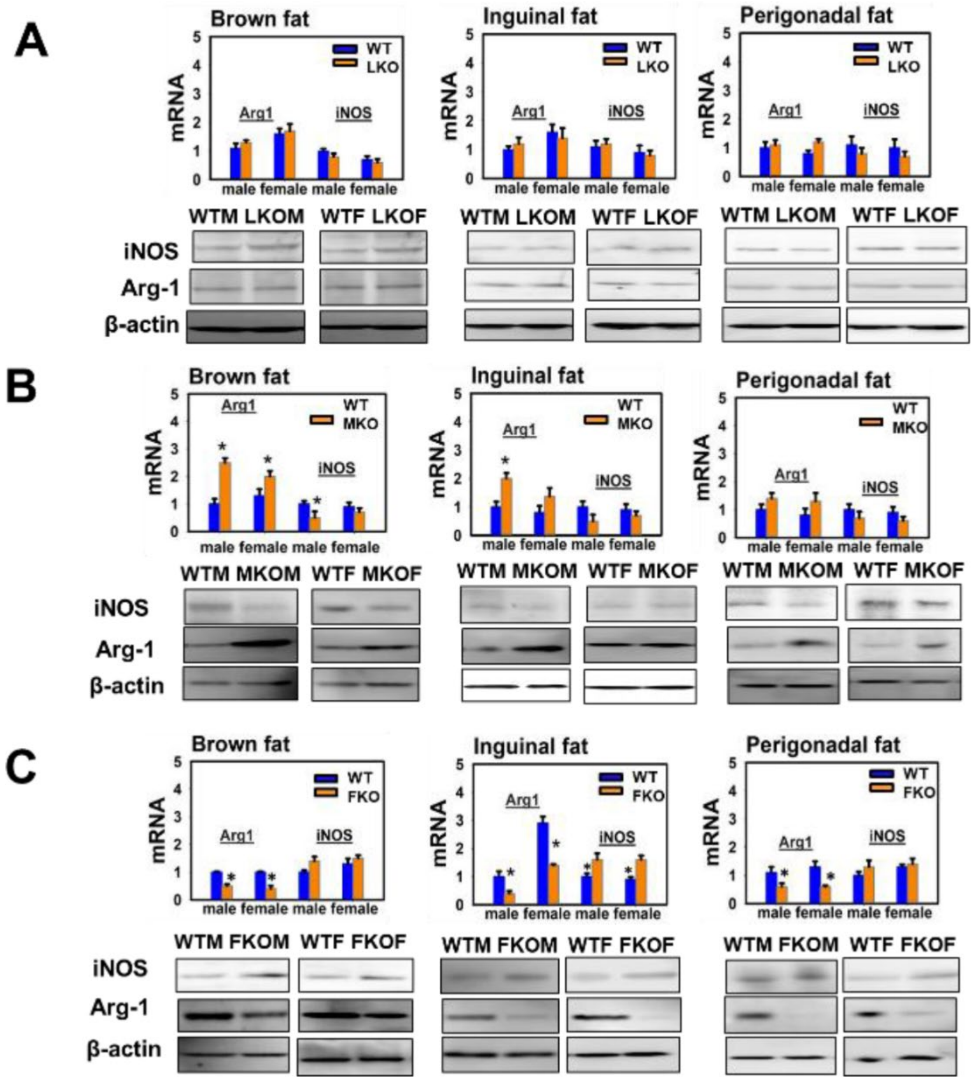

Supplementary Figure 6. Effects of tissue-specific deletion of Growth Hormone Receptor (LKO, MKO, FKO) on adipose tissue macrophage infiltration and macrophage M1-M2 polarization. Quantitative RT-PCR analysis of total RNA isolated from brown fat, inguinal and perigonadal adipose tissues of 24-week-old LKO (**A**), MKO (**B**), FKO (**C**) mice and WT mice for M1 macrophage markers (iNOS) and M2 macrophage markers (Arg1) mRNAs. Data (mean ± SEM; *n* = 4) were normalized by the amount of GAPDH mRNA and expressed relative to the corresponding male WT value. **P* < 0.05, ***P* < 0.01 versus WT. Cell lysate was isolated from brown, inguinal and perigonadal adipose tissues of wt mice and LKO (**A**), MKO (**B**), FKO (**C**). The protein levels of iNOS and Arg1 were measured by western blotting. Relative protein expression was normalized to β-actin levels. Values are mean ±SEM (n = 4).

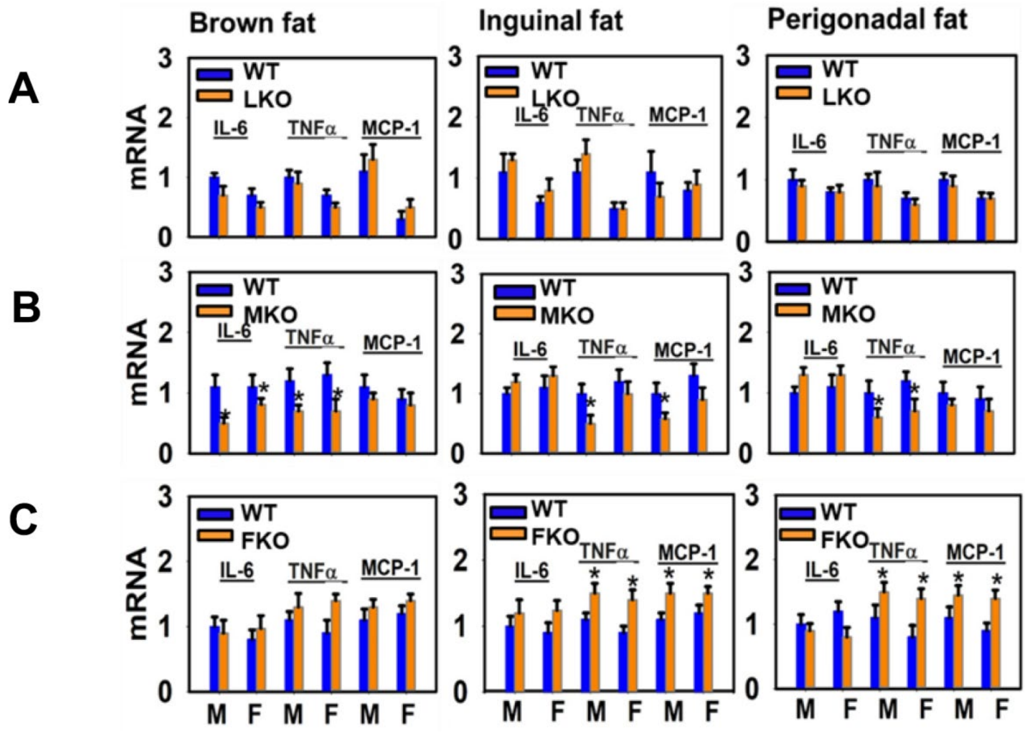

Supplementary Figure 7. (**A**) Quantitative RT-PCR analysis of total RNA isolated from brown fat, inguinal and perigonadal adipose tissues of 24-week-old LKO mice and WT mice for IL-6, TNFα, MCP-1 mRNAs. Data (mean ± SEM; *n* = 4) were normalized by the amount of GAPDH mRNA and expressed relative to the corresponding male WT value. *$P < 0.05$ versus WT. (**B**) Quantitative RT-PCR analysis of total RNA isolated from brown, inguinal and perigonadal adipose tissues of 24-week-old MKO mice and WT mice for IL-6, TNFα, MCP-1 mRNAs. Data (mean ± SEM; *n* = 4) were normalized by the amount of GAPDH mRNA and expressed relative to the corresponding male WT value. *$P < 0.05$ versus WT. (**C**) Quantitative RT-PCR analysis of total RNA isolated from brown fat, inguinal and perigonadal adipose tissues of 24-week-old FKO mice and WT mice for IL-6, TNFα, MCP-1 mRNAs. Data (mean ± SEM; *n* = 4) were normalized by the amount of GAPDH mRNA and expressed relative to the corresponding male WT value. *$P < 0.05$ versus WT.
