# Supplementary material for: Muscle-dependent regulation of adipose tissue function in long-lived growth hormone-mutant mice
Source: Aging (Albany NY). 2020 May 28;12(10):8766–89. doi: 10.18632/aging.103380 (PMC7288969; doi:10.18632/aging.103380)
Supplement: Supplementary Table 1 [file aging-12-103380-s001..pdf]

SUPPLEMENTARY TABLE

Supplementary Table 1. Primer sequences for real-time PCR.

Primer	Sequence
mGAPDH-For	gacaactcactcaagattgtcagcaatgc
mGAPDH-Rev	gtggcagtgatggcatggactgtggtc
UCP1-For	gggcccttgtaaacaacaaa
UCP1-Rev	gtcggtccttccttggtgta
F4/80-For	tgcatctagcaatggacagc
F4/80-Rev	gccttctggatccatttgaa
CD163-For	catgtctctgaggctgacca
CD163-Rev	tgcacacgatctacccacat
CD80-For	ccatgtccaaggctcattct
CD80-Rev	ttcccagcaatgacagacag
TNFα-For	cgtcagccgatttgctatct
TNFα-Rev	cggactccgcaaagtctaag
Arg1-For	cagaacctgctgtcctgtga
Arg1-Rev	tgtcgttggaatcaacctga
iNOS-For	caccttggagttcacccagt
iNOS-Rev	accactcgtacttgggatgc
Il-6-For	agttgccttcttgggactga
Il-6-Rev	tccacgatttcccagagaac
MCP1-For	aggtccctgtcatgcttctg
MCP1-Rev	tctggacccattccttcttg
